# Changes in external ischial tuberosity width at varying trunk–thigh angles between sexes using two measurement methods

**DOI:** 10.1038/s41598-021-99232-w

**Published:** 2021-10-04

**Authors:** Yi-Lang Chen

**Affiliations:** Department of Industrial Engineering and Management, Ming Chi University of Technology, 84 Gung-Juan Road, Taishan, 24301 New Taipei Taiwan

**Keywords:** Anatomy, Engineering

## Abstract

This study examined the influence of two methods and various trunk–thigh (TT) angles on external ischial tuberosity width (EITW) for 45 men and 45 women. In the experiment, the impress and seat pressure methods were applied at TT angles of 60°, 75°, 90°, and 105°. When the impress method was used, EITW remained highly consistent across the four measured TT angles with differences of 2.8 and 2.1 mm for men and women, respectively. Conversely, in the seated pressure method, EITW increased with TT angle such that differences in EITW across a full TT angle range were 11.5 and 11.7 mm for men and women, respectively. Irrespective of method, differences in EITW between genders measured approximately 12.6–13.7 mm across all TT angles. Correlation analyses revealed that hip circumference was positively related to EITW in all cases, whereas the relationship of hip width and depth with EITW varied by method and gender. Because of inherent differences in EITW between genders, these findings suggest that gender variability should be considered in seat cushion design.

## Introduction

Many jobs require prolonged sitting, which may cause harm to the body, lower back and limb pain, and even injury. Accordingly, sitting has received substantial research attention^1,2^. Researchers seek to reform sitting posture and design seating to reduce pain and injury^3–5^. In the study of the effects of sitting on the human body, the contact surface between the buttocks and seat presents a critical inquiry.

**Figure 1:**
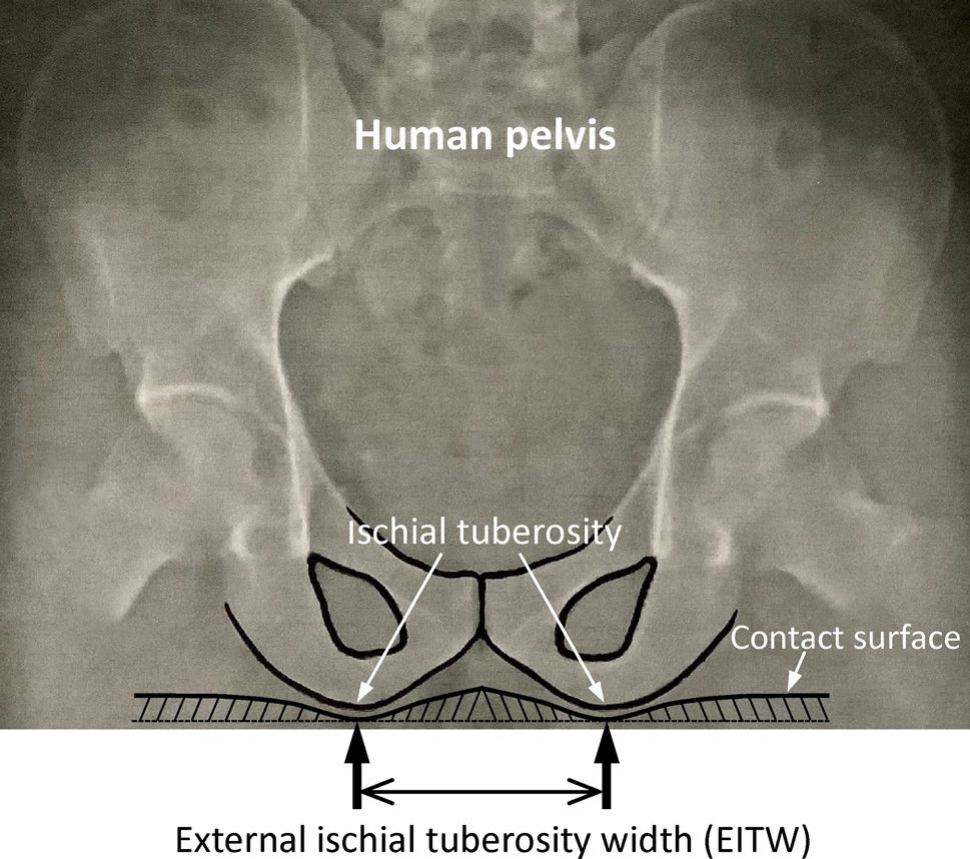
Schematic of the external ischial tuberosity width (EITW).

Seats with different widths and contours produce varying seated pressure distributions, which are mainly influenced by the degree of seated pressure between the ischial tuberosities (ITs) of the human pelvis and the seat surface^6^. Generally, X-ray is the most effective tool for measuring the distance between human ITs^7^. The ITs provide the primary support for the body weight during sitting, however, they do not have direct contact with the seat surface because they are separated by the muscle and adipose tissue that cover the pelvis^8–11^. The buttocks comprise the gluteus maximus and medius muscles, on which a layer of adipose tissue is superimposed and enable primates to sit comfortably. Studies have focused on the complex changes in these subcutaneous tissues between the IT and the seat surface^8–11^. Understanding these changes how to influence the external IT width (EITW, Fig. 1 has practical benefits for the design of seat cushions for bicycles and wheelchairs and in various other contexts^8,10,12,13^.

Magnetic resonance imaging (MRI) is commonly used to identify changes in subcutaneous tissues. Researchers have confirmed that such changes in the buttocks include displacement and deformation^10,11,14–16^, which result from the compression of the chair surface and IT by the sitter’s body weight. Sonenblum et al.^15^ found that changes in subcutaneous tissues are derived from a combination of muscle displacement and distortion during loaded sitting. In an unloaded position, the gluteus and hamstrings overlapped beneath the pelvis, whereas the hamstrings moved anteriorly and the gluteus moved posterolaterally when sitting. Sonenblum et al.^10^ further noted that bulk tissue thicknesses at the ischium, which rarely included muscle, were reduced by more than 60% in enveloping cushion designs and diminished more variably (23%–60%) in an orthotic offloading design. Adipose was typically displaced posteriorly and superiorly from the unloaded condition and resulted in increased lateral displacement. Therefore, the IT is critical to considerations of envelopment or pressure redistribution in seat design^10^. This study assumed that changes in the subcutaneous tissues due to the interaction of ITs with the seat surface may alter the EITW during sitting.

Few studies have included EITW measurement, the seated pressure method^12,17^, and the impress method^6,17–19^. Using the seated pressure method, Chen and Yang^17^ concluded that male EITW measured 134.2 mm, which was considerably wider than Bressel and their colleagues’ findings of 124.0 mm^12^. Using the impress method, Sauer et al.^19^ demonstrated that the EITW measurements of men and women were 116.5 and 134.9 mm, respectively, with a gender difference of 18.4 mm. Similarly, Chen and Yang^17^ reported that the EITW measurements of men and women were 119.6 and 135.2 mm, respectively, with a gender difference of 15.6 mm. These results are consistent with the inherent IT differences between genders.

Because pelvic shape is one of the most substantial differences in geometric morphometrics between men and women^20^, research on EITW gender differences have practical applications for seat design. Compared with men, women have more subcutaneous fat, which is distributed over the buttocks and thighs. Similarly, skeletal differences are especially evident in the pelvis, which has a larger inlet and outlet to accommodate childbirth^21,22^. Furthermore, MRI research limits studies to a single position, and the human body’s trunk–thigh (TT) angle does not remain constant at 90° when interacting with the seat. To our knowledge, no study has conducted EITW measurements at various TT angles. This study, therefore, recruited 45 male and 45 female participants to identify the effects of gender and method variables on EITW values at various TT angles. We hypothesized that gender, TT angle, and measurement method could affect the EITWs and the results can be a reference for seat cushion design.

## Methods

### Participants

A total of 90 healthy participants (45 males and 45 females) were recruited for the study, and they were paid hourly. Individuals with medical histories of musculoskeletal disorders were excluded. Each participant was thoroughly informed of relevant details and experimental procedures. The mean (standard deviation) age, height, and body weight for the men were 21.5 (2.6) years, 172.1 (3.9) cm, and 66.2 (7.3) kg, respectively, and those for the women were 21.7 (2.4) years, 160.5 (3.3) cm, and 52.4 (6.4) kg, respectively. Detailed anthropometric data for the two participant groups are listed in Table 1. To prevent bias caused by extreme body weight and height, and thus affect the results, this study compared participants’ anthropometric data with those from the study of Wang et al.^23^, which focused on young men and women in Taiwan. Data indicated that participants’ anthropometric data were in normal ranges. Informed consent was obtained from all participants and attested for publication of the identifying information/images in an online open-access publication. The experiment was performed in accordance with the 2013 World Medical Association Declaration of Helsinki. The Ethics Committee of Chang Gung Medical Foundation approved the experimental procedures.

**Table 1:** Anthropometric data of the male and female participants.

Items	Males (N = 45)		Females (N = 45)	
	Mean	SD	Mean	SD
Age (years)	21.5	2.6	21.7	2.4
Height (cm)	172.1	3.9	160.5	3.3
Body weight (kg)	66.2	7.3	52.4	6.4
Hip width (cm)	32.8	2.4	33.3	2.9
Hip depth (cm)	22.2	1.9	19.4	2.5
Hip circumference (cm)	93.4	6.1	92.6	6.8

The measurements of hip-related anthropometric data conformed to the methods adopted by Gordon et al.^24^. Hip depth was defined as the horizontal distance between the foremost protruding point of the front and back of the buttocks on the sagittal plane,hip width was defined as the horizontal distance between the foremost protruding points of the hips on the front plane; and hip circumference was defined as the horizontal circumference of the foremost protruding part of the buttocks. The results are shown in Table 1 and are similar or slightly smaller than those of Gordon et al.^24^.

### EITW measurement methods

EITW measurements of the participants were conducted using the impress and seated pressure methods. The measurement of each method was repeated to ensure intrasubject reliability, and the mean of the repeated measurements was calculated for analysis.

**Figure 2:**
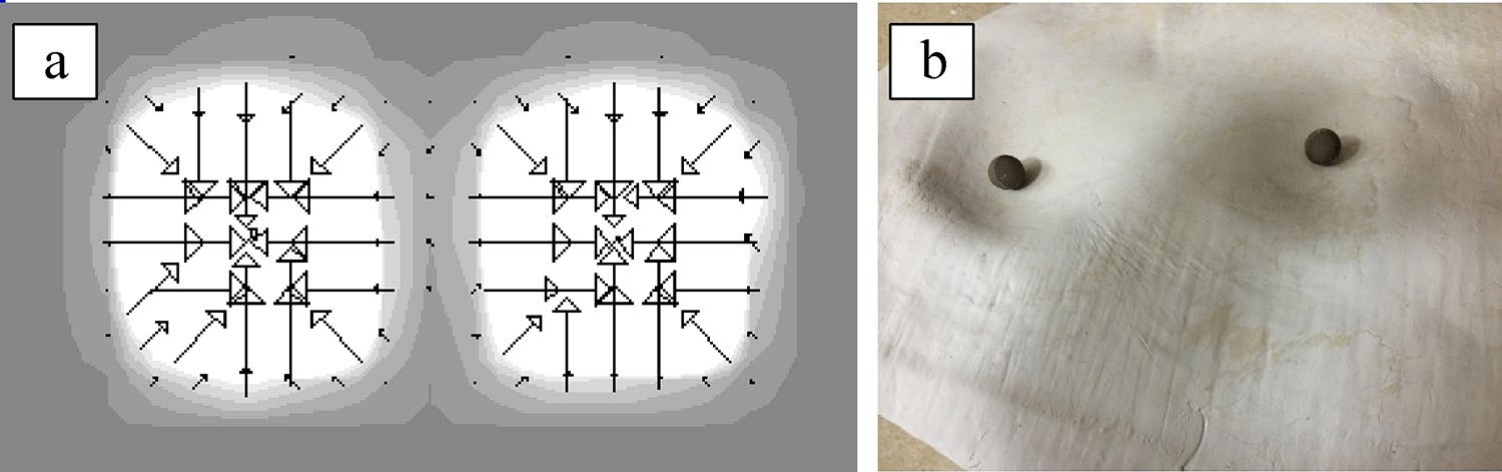
External ischial tuberosity width (EITW) estimation in the seated pressure method (**a**) and the complete EITW imprint in clay in the impress method (**b**).

Conducting measurements using the seated pressure method is a relatively simple procedure commonly employed by researchers^17,25,26^. The mFLEX pressure mapping system (Type 5E, Vista Medical LTD., Netherlands) was adopted for pressure measurement Fig. 2a. The mapping area of the mFLEX system was 0.53 × 0.53 cm^2^, and the length and width of a single sensor was 1.656 and 1.656 cm, respectively, which was converted on a computer by its proportional ratio. Because the maximum measurement pressure was 200 mmHg, the gradient mode of the system was used to calculate the pressure distribution center Fig. 2a, and the EITW values were obtained accordingly.

To implement the impress method, this study revised the methods used by Potter et al.^6^, Chen and Yang^17^, and Chen^18^. The measurement procedure was as follows: (1) A large round plastic basin was placed on the ground,(2) The bottom of the basin was filled with clay, and the surface of the clay was made even and smooth,(3) The clay was covered with a layer of plastic wrap to maintain its moisture and malleability,(4) The participants slowly sat on the clay, and the shape of the participant’s ITs was fully imprinted in the clay Fig. 2b and (5) The participants were instructed to carefully stand up, after which the researcher examined the two concavities in the clay and marked the bottom of each concavity with a small steel ball. The distance between the two balls was measured using a vernier caliper and recorded for analysis.

### Experimental design and procedure

**Figure 3:**
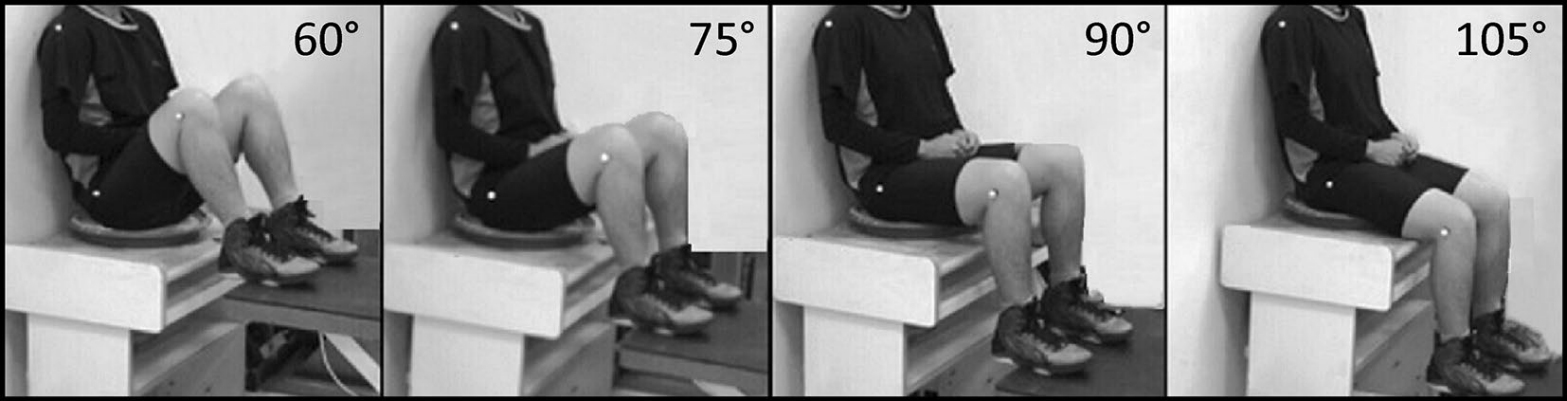
Four varying trunk–thigh angles adopted in the study.

Data from our trials (2 methods × 4 TT angles × 2 repetitions) were collected for 45 male and 45 female participants. A total of 1440 EITW data were collected. The study used two measurement methods: the impress and seat pressure methods. Participants were requested to perform the described postures at four TT angles Fig. 3, namely, 60°, 75°, 90°, and 105°, which are commonly adopted angles during daily sitting^27,28^. During the experiment, the participants were required to wear sportswear and very thin, elastic, and tight pants. Sportswear was provided by the participants or the researchers. To ensure accurate TT positions, three adhesive reflective markers were attached to a participant’s dominant-side acromial shelf, greater trochanter, and lateral epicondyle prior to data collection Fig. 3. A lifting platform adjustable upwards and downwards was used to set the appropriate TT angles. Before testing, the participants were asked to adjust their sitting postures as slowly as possible while looking straight ahead with their backs resting against the wall and hands hanging or clasping naturally. As described by Chen et al.^29^, a researcher adjusted the height of the platform to match the specific TT angle with a preset line on the feedback monitor. The measurement sequence for the participants was in a random order.

### Statistical analyses

The EITW measurements obtained through both methods were analyzed using SPSS Version 23.0 (IBM Corp., Armork, NY, USA) with a significance level set to α = 0.05. A three-way repeated-measures analysis of variance (ANOVA) was used to examine the effects of the gender, measurement method (i.e., impress and seated pressure), and TT angle variables on EITW. Moreover, four one-way ANOVAs for each gender (2) × posture (2) combination were conducted for cross-analyses. Duncan’s multiple range test (MRT) was used for post hoc analysis. A power value was used to determine whether the effect size of any significant independent variable was satisfactory^30^. The Shapiro–Wilk test was used to assess whether the dependent variables were normally distributed, and the Pearson product–moment correlation (r) was used to ensure the intrasubject reliability of the repeated measurements and correlations between anthropometric data and the EITW for each test combination.

## Results

### Three-way ANOVA results

The reliability of the repeated measurements of each test combination, irrespective of gender, exceeded 0.858 (all *p *< 0.01), which indicated sufficient consistency. The three-way ANOVA results indicated that gender, measurement method, and TT angle variables all significantly affected EITW (all *p *< 0.001). The interaction term of method × TT angle was also significantly affected (*p *< 0.001) Table 2, which suggested that cross-analyses were needed to clarify the interaction effects.

**Table 2:** Main and interaction effects of external ischial tuberosity widths obtained from three-way ANOVA.

Sources	DF	SS	MS	F	*p*	Power
Gender (G)	1	12,323	12,323	108.2	< 0.001	1.000
Method (M)	1	2998	2998	26.3	< 0.001	0.999
Trunk-thigh angle (TTA)	3	2250	7501	6.6	< 0.001	0.973
G × M	1	1	1	< 0.1	0.914	0.916
G × TTA	3	10	3	< 0.1	0.993	0.051
M × TTA	3	5170	1723	15.1	< 0.001	1.000
G × M × TTA	3	14	5	< 0.1	0.989	0.057

### Cross-analyses of one-way ANOVA

**Figure 4:**
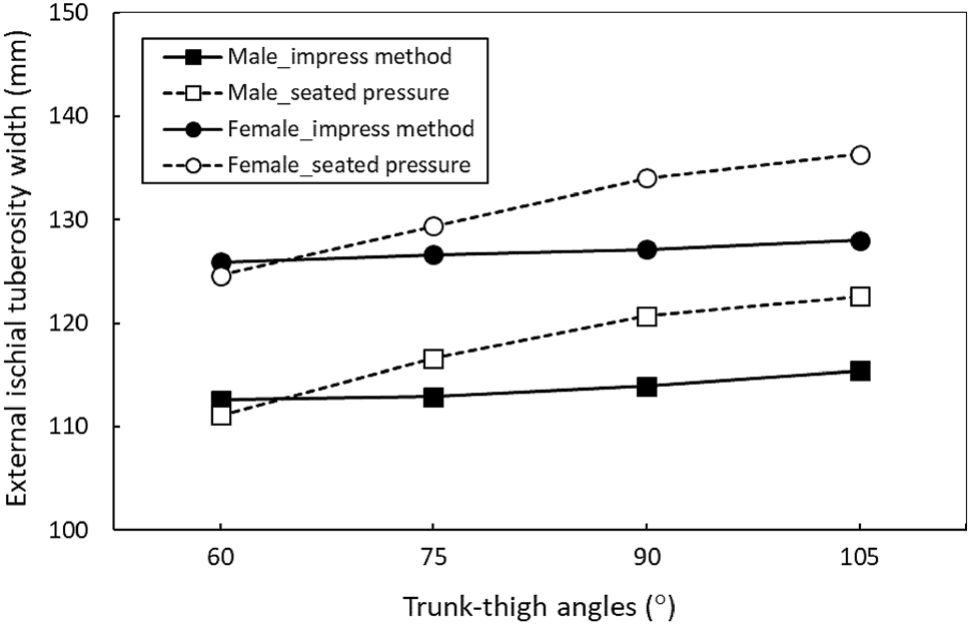
Comparisons of external ischial tuberosity width values under different testing conditions.

Table 3 shows the results of the four one-way ANOVAs for the varying gender and method combinations. According to these findings, the seated pressure method resulted in different EITW values for the measured TT angles (all *p* < 0.001), whereas the impress method did not result in such variations. The Duncan MRT results indicated that as the TT angle increased, the EITW using the seated pressure method also increased with ranges of 111.1–124.6 mm at 60° and 122.6–136.3 mm at 105° for men and women, respectively Table 4. Figure 4 indicates that the EITW values varied with the TT angles across the four gender and method combinations. Irrespective of gender, the EITW values measured using the seated pressure method increased as the TT angle increased, whereas the values measured using the impress method remained unchanged at all TT angles.

**Table 3:** Main effects of trunk–thigh angle on external ischial tuberosity width measurement.

Genders	Methods	F	*p*	Power
Male	Impress	1.0	0.379	0.278
	Seated pressure	6.5	< 0.001	1.000
Female	Impress	0.3	0.806	0.113
	Seated pressure	11.6	< 0.001	0.999

**Table 4:** Duncan’s multiple range test results on external ischial tuberosity width for one-way ANOVA.

Trunk-thigh angle	Impress (mm)		Seat pressure (mm)	
	Males	Females	Males	Females
60°	112.6 (9.0) a*	125.9 (8.4) a	111.1 (12.7) a	124.6 (10.9) a
75°	112.9 (7.8) a	126.6 (10.3) a	116.6 (12.9) ab	129.4 (10.2) b
90°	113.9 (7.8) a	127.1 (11.4) a	120.7 (13.4) bc	134.0 (8.7) c
105°	115.4 (8.0) a	128.0 (10.0) a	122.6 (13.9) c	136.3 (10.5) c

^*^Data (mean, with standard deviation in parentheses) with the same lowercase letter do not differ in the Duncan test.

### Correlations between anthropometric data and EITW values

Table 5 shows the correlation analyses between hip-related dimensions and the EITW values. The results indicated that hip circumference was positively correlated with all EITW values. Nevertheless, correlations of hip width and depth with EITW varied across gender and method variables. In the impress method, hip depth was related to EITW for both genders, whereas hip width was a more significant indicator for men.

**Table 5:** Correlation coefficients (r) between external ischial tuberosity width and three hip dimensions for various test combinations.

TT angle	Impress				Seated pressure			
	60°	75°	90°	105°	60°	75°	90°	105°
**Male**								
Hip width	0.558***	0.459**	0.317*	0.365*	0.276	0.201	0.235	0.358*
Hip depth	0.623***	0.531***	0.336*	0.377*	0.167	0.225	0.327*	0.337*
Hip circumference	0.688***	0.589***	0.351*	0.344*	0.326*	0.357*	0.401**	0.453**
**Female**								
Hip width	0.250	0.265	0.270	0.162	0.048	0.123	0.281	0.229
Hip depth	0.323*	0.330*	0.357*	0.464**	0.220	0.013	0.119	0.230
Hip circumference	0.335*	0.376*	0.485***	0.342*	0.354*	0.388*	0.341*	0.437**

*p < 0.05; **p < 0.01; *** *p *< 0.001.

## Discussion

This study evaluated the influences of gender, measurement methods, and TT angles on the EITW of 45 male and 45 female participants. EITW has practical application for seat cushion designs such as those in wheelchairs and bicycles. This study found that all variables significantly impacted EITW. Notably, EITW was greater for women than for men. EITW measured through the seated pressure method increased as TT angle increased, whereas measurement through the impress method resulted in no significant changes on EITW.

Applying the seated pressure method, the male EITW were 124 and 134.2 mm measured by Bressel et al.^12^ and Chen and Yang^17^, respectively. EITW in this study ranged from 111.1 at a 60° TT angle to 122.6 mm at a 105° TT angle. The differences in results across studies may in part be attributed to varying TT angles adopted for measurement. Chen and Yang^17^ set the TT angle to 135°, which may have resulted in a larger EITW. Similarly, Sauer et al.^19^ concluded that the difference in EITW between genders was approximately 18.4 mm when the impress method was used, whereas Chen and Yang^17^ reported a difference of 15.6 mm. In the present study, the gender difference was 12.6–13.7 mm Table 4, Fig. 4, which is slightly smaller than that reported in past research. Notably, unlike the TT angles measured in the current study, only a fixed TT angle has been employed in past research. These findings on the differences in EITW between genders may assist in seat cushion design optimization.

EITW measurement has typically included the impress method^17–19^ and the seated pressure method^12,17^. The impress method requires participants to sit on soft clay, whereas the seated pressure measurement is conducted on a flat solid surface. This may influence EITW and explain why EITW did not vary at differing TT angles in the impress method. Soft materials may mostly reflect IT on EITW. Al-Dirini et al.^14^ found that the subcutaneous soft tissues around the IT become spatially distorted below the ischium along the thigh in the distal direction. This may cause that the EITW increased with TT angle increase. However, the conjecture should be further clarified, such as examined using MRI.

The correlation analyses Table 5 indicated that hip circumference was positively correlated with EITW under all test conditions (r ranged from 0.326, *p *< 0.05–0.688, *p *< 0.001). Anatomically, the IT and hip width appear to have a morphometric correlation^31^. However, hip width did not predict women’s EITW and was only correlated with men’s EITW under the impress method. However, correlation between hip depths and EITW applied to both genders under the impress method. Chen^18^ found that hip circumference was the most effective predictor of male and female EITW under the impress method. Our results confirmed Chen’s findings. However, the TT angle was set to 135° in Chen’s study, whereas the angles in the present study ranged from 60° to 105°, indicating that correlations of hip circumference with EITW are still valid at smaller TT angles. Because EITW is affected by changes in subcutaneous tissues, two-dimensional hip size (i.e., hip circumference) may reflect alterations in EITW measurement more accurately than one-dimensional hip width or depth can. This study also revealed that the correlation coefficients between hip circumference and hip width × depth of male and female participants were 0.962 and 0.895, respectively, which suggests that a single hip width or depth measurement cannot completely predict EITW. This study attempted to clarify the correlations between the EITW and participant’s body weight and height, however, the correlations were hardly significant. One possible reason is that, although pelvis size and changes in subcutaneous tissues of buttocks may be related to the body size of an individual, the EITW values were predominately influenced by the pelvis geometry. Even so, the hip circumference was more relevant to the EITW than hip width and depth as found in the study.

The present study has several limitations. First, all 90 participants were young people within a normal anthropometrical range. Because obesity is positively correlated with pelvis size^32^, the EITW measurements may have been affected. Moreover, differences in subcutaneous tissue changes exist between participants with and without spinal cord injury when sitting^11^. The usefulness of the findings for other populations (e.g., older people) is also a matter for further investigation. The limitations of this study should be considered in future applications of the results.

## Conclusion

This study employed two measurement methods to collect and compare the EITW values of 90 participants at four TT angles. The results indicated that, irrespective of method, the EITW was approximately 13 mm larger for women than for men. In the seated pressure method, the TT angle resulted in varying EITWs, whereas EITWs were relatively unaffected by TT angle in the impress method. Additionally, hip circumference was correlated with EITW under all test conditions. Findings from this study may serve as a reference for supportive body weight positions in seat cushion designs.
